# Transcriptional profiling identifies IL-33-expressing intestinal stromal cells as a signaling hub poised to interact with enteric neurons

**DOI:** 10.3389/fcell.2024.1420313

**Published:** 2024-08-01

**Authors:** Patrycja M. Topczewska, Anna Savvopoulou, Catalina Cosovanu, Christoph S. N. Klose

**Affiliations:** Department of Microbiology, Infectious Diseases and Immunology, Corporate Member of Freie Universität Berlin and Humboldt-Universität Zu Berlin, Charité—Universitätsmedizin Berlin, Berlin, Germany

**Keywords:** IL-33, stromal cells, neuro-immune axis, VIP (vasoactive intestinal peptide), type 2

## Abstract

Recent advancements in mucosal immunology have unveiled a complex network of intercellular connections within diverse tissues, shedding light on the unique properties of different cell types. Central to this intricate network is the cytokine IL-33, which has gained significant attention for its critical role in various diseases, from allergy to cancer, triggering type 2 immune responses, among others. Recent research has challenged the prior assumptions attributing IL-33 expression to epithelial cells, highlighting stromal cells as the predominant source in adipose tissue and the lungs. However, in the complex landscape of the intestine, where IL-33 plays a crucial role in mediating immune surveillance and tolerance and is implicated in many gut-related disorders, its primary source, regulation, and main characteristics need more exploration. This study identifies stromal cells as the primary IL-33-expressing cell type in the small intestine. By investigating their transcriptome and intrinsic signaling pathways, we have uncovered a possible role of IL-33^+^ stromal cells in maintaining the stem cell niche and their potential crosstalk with neurons relevant to the regulation of axonogenesis. Importantly, our experiments have demonstrated that vasoactive intestinal peptide stimulation of a primary intestinal stromal cell culture significantly amplifies IL-33 expression on mRNA and protein level. Therefore, our study represents a significant leap forward in understanding the plethora of interactions IL-33^+^ intestinal stromal cells maintain in the intestine, paving the way for future investigations into stromal-neuro crosstalk in the gut. These findings hold great promise for developing targeted therapeutic strategies aimed at harnessing the potential of IL-33 across a spectrum of diseases.

## Introduction

Interleukin-33 (IL-33) is a key cytokine, exerting pivotal roles in immune regulation, tissue homeostasis, and the pathogenesis of various diseases (Molofsky et al., 2015). Elevated IL-33 levels are associated with conditions such as asthma, inflammatory bowel disease, cardiovascular disorders, and certain cancers, making it a significant biomarker and therapeutic target (Farshadi et al., 2019; Liu et al., 2022; Wang et al., 2023a; Pan et al., 2023; Zhao et al., 2023). In recent years, IL-33 expression by stromal cells has emerged as a topic of intense investigation, shedding new light on the complex interplay between the immune system and tissue microenvironments (Dahlgren et al., 2019; Mahlakoiv et al., 2019; Rana et al., 2019; Spallanzani et al., 2019). IL-33 primarily localizes in the nucleus and can act as an alarmin when released during cell death or inflammation. Its receptor, ST2, is found on ILC2s, Tregs and Th2 cells, but also granulocytes and DCs, triggering type 2 immune responses (Moro et al., 2010; Neill et al., 2010; Hung et al., 2013; Turner et al., 2013; Molofsky et al., 2015; Jarick et al., 2022; Topczewska et al., 2023). In parallel, stromal cells have emerged as key players in tissue microenvironments, with diverse functions beyond structural support. Recent studies propose that they are the main source of IL-33 in adipose tissue, the lungs, and the colon (Dahlgren et al., 2019; Mahlakoiv et al., 2019; Rana et al., 2019; Spallanzani et al., 2019; Waddell et al., 2021). In the intestine, IL-33 expression has been detected in various cell types, whereas its expression in subepithelial mesenchymal cells, specifically stromal cells, remains understudied, and the precise phenotype of these stromal cells is yet to be fully characterized (Furukawa et al., 2017; He et al., 2018; Hung et al., 2020; Wang et al., 2023a). In this manuscript, we demonstrate that intestinal PDGFR-α^+^ Sca-1^+^ stromal cells are the primary producers of IL-33 and capable to interact with ILC2. Through transcriptional profiling of IL-33-eGFP^+^ and IL-33-eGFP^−^ stromal cells, we dissect possible molecular interactions with various cell types and elucidate their potential role in axon guidance and axonogenesis within the intestine. Additionally, we provide evidence that the neuropeptide vasoactive intestinal peptide (VIP), acting on the Vipr2 receptor expressed by stromal cells, positively regulates IL-33 expression in these stromal cells. These findings underscore the intricate interplay between stromal cells, IL-33, and neuronal elements in intestinal physiology and pathology, offering novel insights into the mechanisms underlying intestinal homeostasis and inflammation, and suggesting new pathways for therapeutic intervention in intestinal inflammatory disorders.

## Results

### PDGFR- α ^+^ Sca-1^+^ stromal cells are the main IL-33^+^ cell type in the small intestine

**FIGURE 1:**
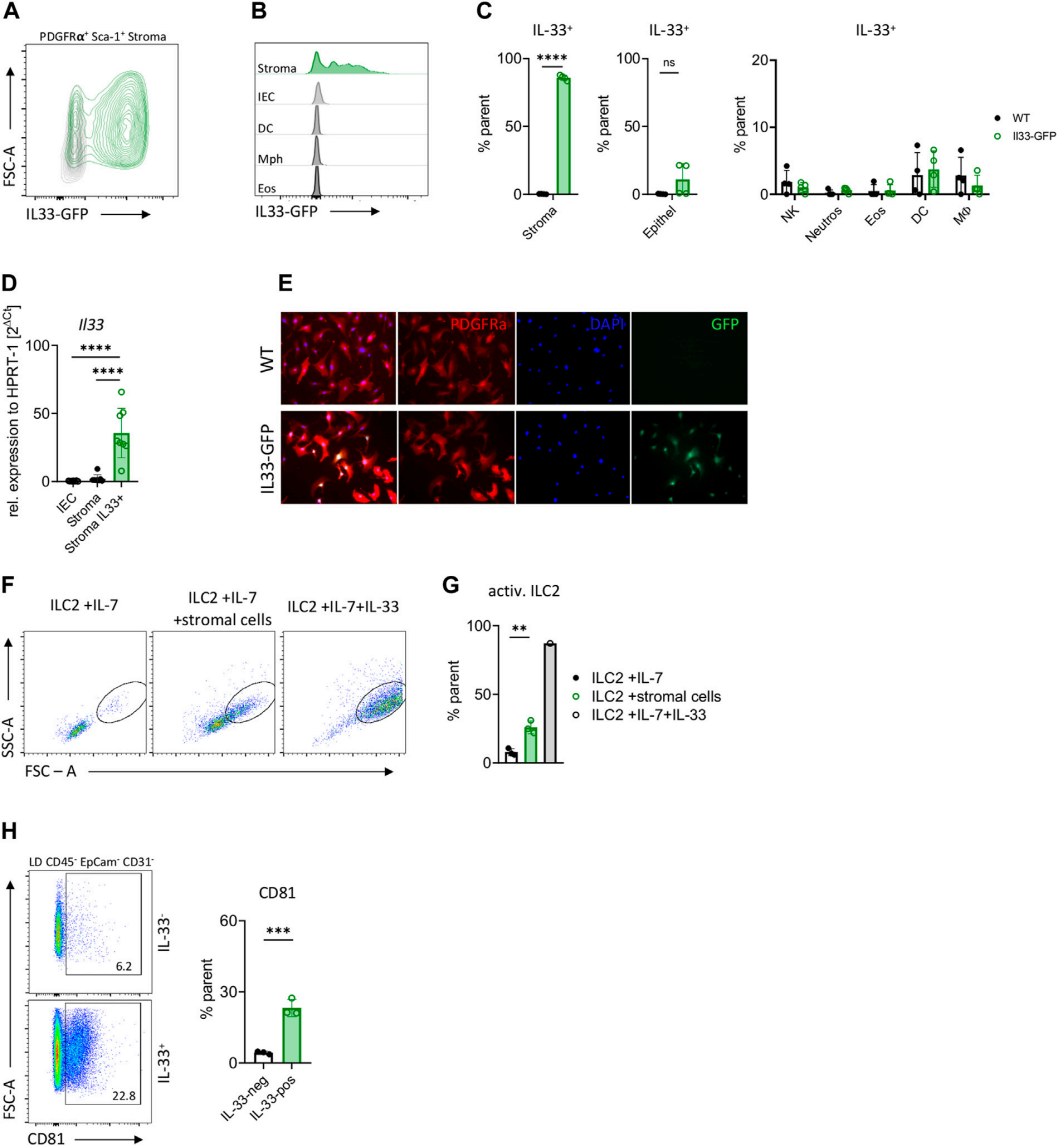
*Il33* is mainly expressed by intestinal stromal cells. **(A)** Flow cytometric plot of intestinal stromal cells from IL-33-eGFP reporter mice. **(B)** Flow cytometric histograms of IL-33 expression in intestinal stromal cells, intestinal epithelial cells (IEC), dendritic cells (DC), macrophages (Mph) and eosinophils (Eos) from IL-33-eGFP reporter mice. **(C)** Percentage of IL-33 expression from **(B)**, with addition of neutrophils and NK cells. **(D)**, Expression of *Il33* gene in sort-purified intestinal epithelial cells (EpCAM^+^), intestinal IL-33-eGFP^-^ stromal cells and IL-33-eGFP^+^ stromal cells (PDGFR- α ^+^, Sca-1^+^) from IL-33-eGFP reporter mice. **(E)** Immunofluorescence of sort-purified IL-33-eGFP^+^ cultured stromal cells. IL-33 expression is represented by eGFP reporter expression in green. **(F)** Flow cytometric plots of small intestine ILC2s from WT mice after co-culture with IL-7, IL-7 and stromal cells, or IL-7 and IL-33. ILC2s were gated on live CD45^+^ Lin^−^ (CD3, CD5, CD19, Ly6g, and Fcer1), CD127^+^ and KLRG1^+^. **(G)** Evaluation of ILC2 activation from F after 3 days in culture with cytokines IL-7 or IL-7 and stromal cells or IL-7, IL-33. **(H)** Flow cytometric plots and evaluation of CD81 expression of small intestine stromal cells from IL-33-eGFP mice. Stromal cells were gated on LD, CD45^−^, PDGFR-α^+^ and IL-33^+^ or IL-33^−^. **(C, D, G, H)**: Each symbol represents data from 1 mouse, data are representative of two experiments with three to four mice per group. Mean±SD, Student’s *t*-test or one-way ANOVA. ns not significant, **p < 0.01, ***p < 0.001, ****p < 0.0001.

We employed different methods in order to unequivocally examine the source of the IL-33 cytokine in the small intestine. Our investigation, utilizing IL-33-eGFP mice and WT controls, revealed that both IL-33 protein and *Il*33 mRNA expression were predominantly observed in stromal cells rather than epithelial or immune cells (Figures 1A–D; Supplementary Figure S1). Nearly all intestinal stromal cells were found to be IL-33-eGFP^+^ positive, whereas only half of the inspected mice showed IL-33 expression in epithelial cells, being around 10% (Figures 1A–C). Although IL-33 was shown to be expressed by macrophages and DC under certain conditions (Furukawa et al., 2017; Wang et al., 2018; Hung et al., 2020), we failed to detect IL-33 expression over background in the small intestinal immune cells investigated from naïve mice (Figure 1C). To further elucidate the localization of IL-33 within stromal cells, we sort-purified these cells from the small intestine of IL-33-eGFP mice and cultured them until reaching confluence. Immunohistochemistry showed the presence of IL-33 cytokine mainly in the nucleus but also in the cytoplasm of stromal cells (Figure 1E). Additionally, co-culture experiments with sort-purified ILC2s and small intestine stromal cells demonstrated an enhanced blasting of ILC2s compared to those cultured with IL-7 alone, albeit not reaching the same level as those cultured with recombinant cytokines (Figures 1F,G). To better define IL-33^+^ stromal cells, we conducted phenotypic characterization of PDGFR-α^+^ Sca-1^+^ IL-33-positive and IL-33-negative cells using flow cytometry. Both populations uniformly expressed the markers CD90, CD49a, and Podoplanin consistent with previous results (Waddell et al., 2021) (Supplementary Figure S2A,B). A fraction of IL-33^+^ cells expressed the marker CD81 that was barely detectable in IL-33^-^ stromal cells (Figure 1H; Supplementary Figure S2C). These findings collectively indicate that stromal cells are the primary source of IL-33 in the small intestine and have a potential role in ILC2 activation, consistent with previous studies in adipose tissues and lungs (Dahlgren et al., 2019; Mahlakoiv et al., 2019).

### IL-33-expressing intestinal stromal cells support epithelial crypts and intestinal innervation

**FIGURE 2:**
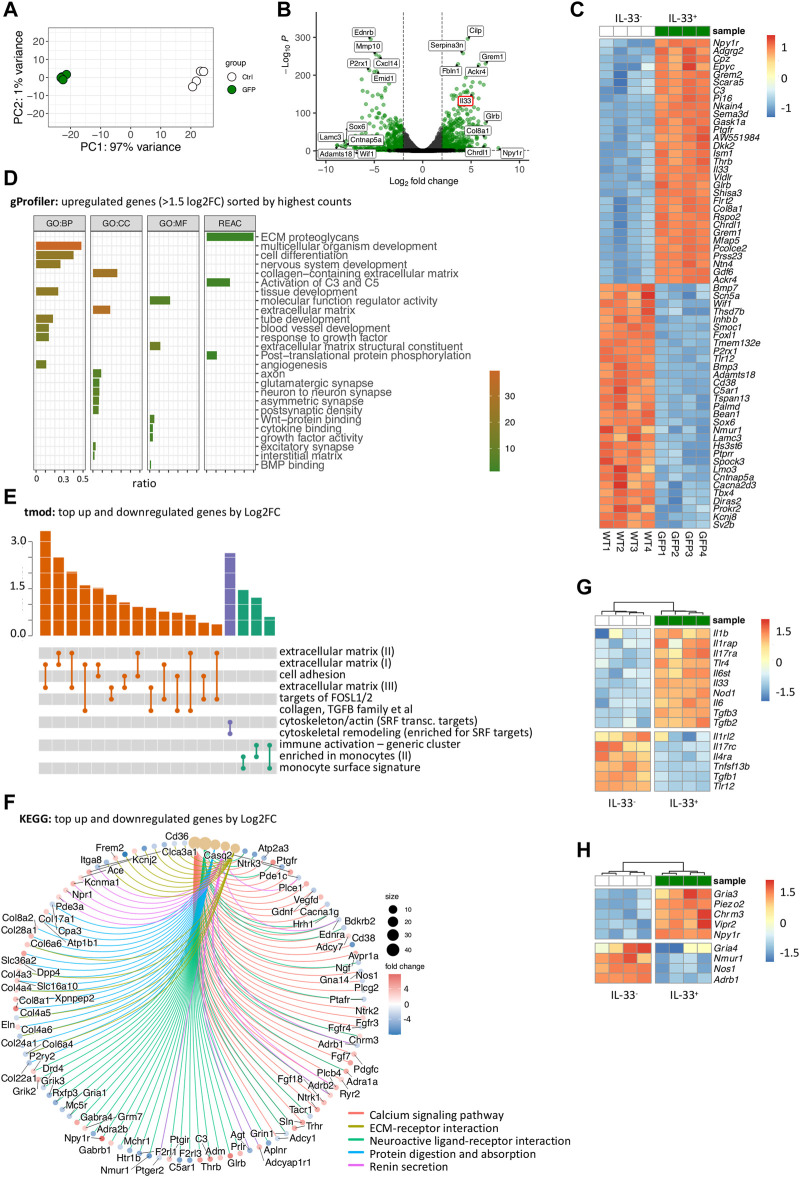
Transcriptional profiling of IL-33-GFP^+^ stromal cells reveals a plethora of molecular interactions with immune cells, neurons, epithelial cells and the extracellular matrix **(A)** Principal component analysis of RNA-Seq of IL-33-eGFP^−^ and IL-33-eGFP^+^ stromal cells. **(B)** Volcano plot of RNA-Seq from **(A)** showing differentially expressed genes and upregulation of *Il33*. **(C)** Heatmap of the top 30 up- and down-regulated genes (by log2FC) in IL-33-eGFP^+^ and IL-33-eGFP^−^ intestinal stromal cells. **(D)** Top upregulated pathways after gProfiler gene ontology analysis of significantly differentially expressed genes with a Log2FC > 1.5 and sorted by BaseMean (counts) of IL-33-eGFP^+^ and IL-33-eGFP^−^ intestinal stromal cells. **(E)** Top pathways after tmod gene ontology analysis of significantly differentially expressed genes of IL-33-eGFP^+^ and IL-33-eGFP^−^ intestinal stromal cells. **(F)** Top pathways after KEGG gene ontology analysis of significantly differentially expressed genes of IL-33-eGFP^+^ and IL-33-eGFP^−^ intestinal stromal cells. **(G,H)** Heatmaps of immune related genes **(G)** and neuron related genes **(H)**.

To determine the primary function and unique attributes of IL-33-expressing stromal cells in the small intestine, we performed bulk RNA-sequencing on PDGFR- α ^+^ Sca-1^+^ intestinal stromal cells, comparing IL-33^+^ (IL-33-eGFP^+^) to IL-33^-^ (IL-33-eGFP^-^) cells using IL-33-eGFP reporter mice. Initially, the principal component analysis (PCA) revealed distinct clustering of stromal cell populations based on IL-33 expression, indicating significant differences between IL-33^+^ and IL-33^-^ stromal cells (Figure 2A). Consistently, IL-33 expression was markedly higher in IL-33^+^ stromal cells compared to the negative population and top hits involved, e.g., extracellular matrix genes like *Col8a1, Fbln1* in the IL-33^+^ compartment or *Pi16* shown to be enriched in fibroblasts near vascular structures, while in the IL-33^-^ compartment we found genes, such as *Cxcl14* and *Bmp7* already detected in colon and bladder fibroblasts (Muhl et al., 2020) (Figures 2B,C).

Subsequently, we analyzed pathways defined by upregulated (>1.5 log2FoldChange) and highly expressed (sorted by high counts per row mean) genes using gProfiler (Figure 2D; Supplementary Figure S3). Additionally, we identified pathways of all significantly differentially expressed genes using tmod and KEGG databases (Figures 2E,F; Supplementary Figure S4), without considering the counts per row mean. To gain deeper insights into the phenotype of the IL-33^+^ stromal cells, we generated heatmaps of imme-related and neuron-related genes, based on identified pathways (Figures 2G,H). These findings underscore the significant involvement of IL-33-expressing stromal cells in various processes, including extracellular matrix dynamics, tissue development and remodeling, WNT and BMP signaling, complement system activation (C3), and cytokine binding, consistent with previous research (Powell et al., 2011; Roulis and Flavell, 2016; Kinchen et al., 2018; Zheng et al., 2021) (Figures 2D–F). As IL-33 is an alarmin associated with inflammation, we examined differentially expressed genes in an immune-related context. Our analysis suggests a potential involvement of IL-33^+^ stromal cells in immune regulation through co-expression of additional cytokines and receptors, such as *Il1b*, *Il6*, *Il17ra*, *Il31ra* and *Tlr4* (Figure 2G; Supplementary Figure S7A). We also found IL-33^+^ stromal cells expressing antagonists of the bone morphogenetic protein (BMP): *Grem1*, *Grem2* and *Chrdl1*, while also expressing WNT signaling components: *Wnt2*, *Wnt2nb*, *Wnt9* and *Wnt11* (Figures 2B,C). WNT signaling is necessary for the maintenance of non-differentiated and proliferating stem cells while BMP antagonizes WNT signaling inducing differentiation of epithelial cells (Andoh et al., 2002; He et al., 2004; Zhang et al., 2015; Degirmenci et al., 2018). These findings support the idea of IL-33^+^ stromal cells being important for maintaining LGR5^+^ intestinal epithelial stem cells. Additionally, our analysis provides evidence for IL-33^+^ stromal cells to play an essential role in the interaction with the nervous system, as indicated by the pathways “nervous system development,” “axon,” “glutamatergic synapse,” “neuron-to-neuron synapse” interactions, “neuroactive-ligand receptor interaction” (Figures 2D,F; Supplementary Figure S3). Interestingly, we identified *Npy1r* as the most significantly upregulated gene (Figures 2B,C,H). The neuropeptide Y receptor 1 (NPY1R) is a G-protein coupled receptor that binds neuropeptide Y (NPY), a neurotransmitter involved in various physiological processes, including appetite regulation, stress response, and cardiovascular function (Park et al., 2022). Moreover, IL-33^+^ intestinal stromal cells were found to exhibit a high expression of the receptor vasoactive intestinal peptide receptor 2 (VIPR2), which binds the neuropeptide vasoactive intestinal peptide (VIP), connected to type 2 immune responses (Nussbaum et al., 2013; Talbot et al., 2020; Pascal et al., 2022), the regulation of circadian rhythm in the intestine and nutrient uptake.

Taken together, our findings highlight the potential significance of IL-33^+^ stromal cells in supporting LGR5^+^ intestinal epithelial stem cells in the small intestine. Moreover, these stromal cells appear to contribute to tissue remodeling and growth, including neurogenesis and axon guidance, implicated by the expression of semaphorins and slit genes. It would be of interest for future investigations to further dissect the role of the neuropeptide receptors on IL-33^+^ stromal cells in the intestine, as the enteric nervous system plays an important role in regulating homeostasis and inflammation in the gut.

### GO-analysis suggests a neurodevelopmental role for intestinal IL-33-expressing stromal cells

**FIGURE 3:**
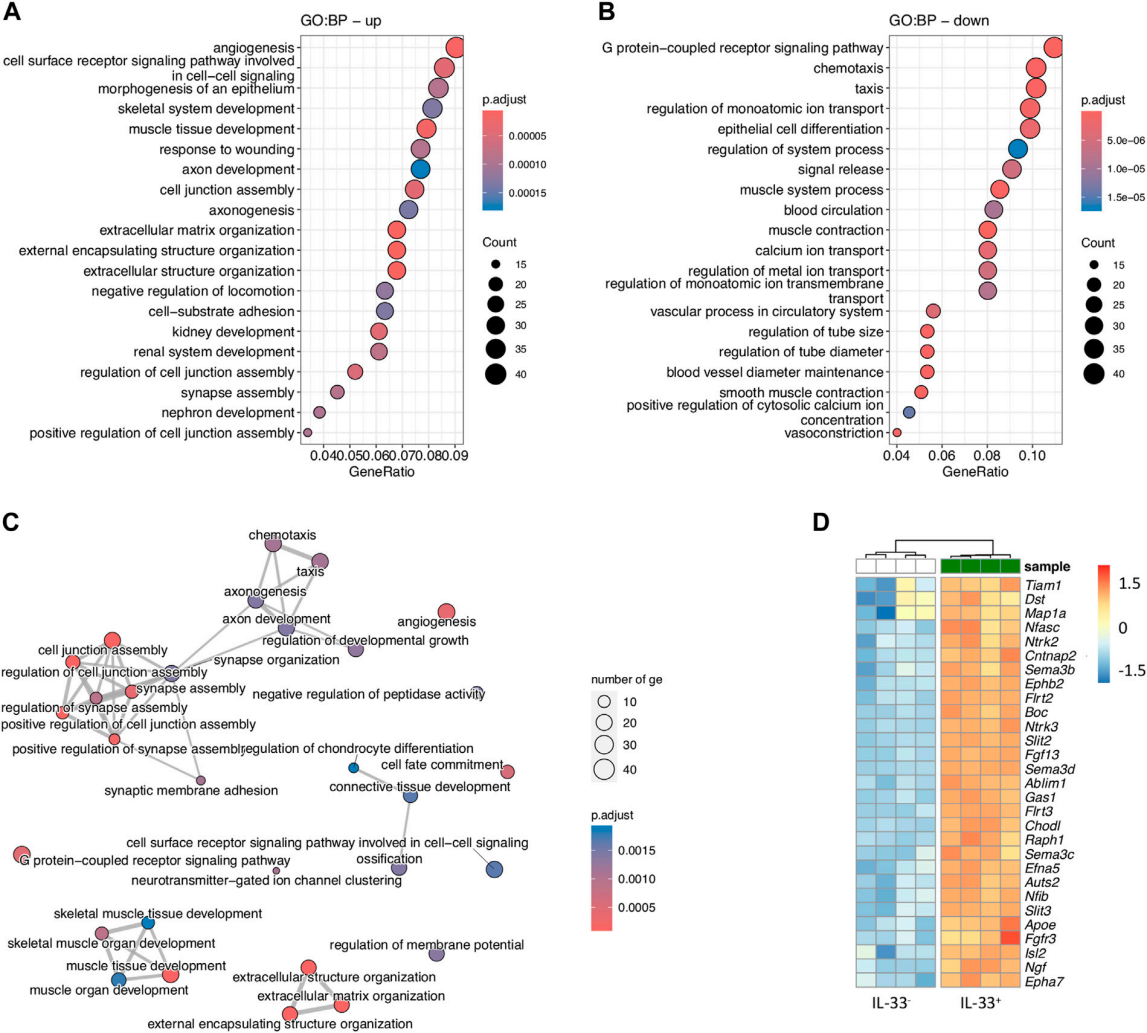
Gene Ontology analysis of IL-33-eGFP^+^ intestinal stromal cells suggests their involvement in axon guidance and **(A, B)** Gene ontology analysis using downregulated **(A)** and upregulated **(B)** genes between IL-33-eGFP^+^ vs. IL-33-eGFP^-^ intestinal stromal cells. **(C)** Netplot showing pathways from **(A)** and **(B)**. **(D)** Heatmap of differentially expressed genes from GO:BP pathway axonogenesis.

To thoroughly examine the role of the differentially expressed genes (DEGs), we expanded our analysis using the Gene Ontology Biological Processes (GO:BP) database separately on upregulated and downregulated genes, comparing IL-33^+^ and IL-33^-^ intestinal stromal cells (Figures 3A–C). The most significant upregulated pathways included, among others, angiogenesis (involving growth factor VEGFD, hematopoietic progenitor cell antigen CD34, angiotensin-I converting enzyme ACE), followed by cell-to-cell signaling and morphogenesis of epithelium, consistent with previous results (Figures 3A–C). Moreover, we found the pathways “axon development” and “axonogenesis” in the top ten upregulated pathways, strengthening the idea of stromal cells being involved in neuronal development. The genes associated with these pathways were, e.g., *Ngf* (encoding nerve growth factor), a well-known neurotrophic factor which plays a crucial role in survival, differentiation and growth in neurons (Ferraguti et al., 2024) (Figure 3D; Supplementary Figures S5,S6). Semaphorins (*Sema3b, Sema3c, and Sema3d*), belonging to a family of guidance cues can repel or attract axons. They are particularly important in the guidance of axons during neuronal development (Koncina et al., 2007). Further, we found slit genes *Slit2, Slit3*, which act as repulsive axon guidance cues. They bind to ROBO receptors on growth cones to regulate axon guidance (Tong et al., 2019) (Figure 3D). Interestingly, IL-33^+^ stromal cells expressed *Ntrk2* and *Ntrk3* (neurotrophic tyrosine kinase receptor two and 3), encoding for a type of receptor tyrosine kinase (RTK) (Figure 3D). In summary, our findings unveil novel insights into the roles of IL-33^+^ stromal cells, shedding light on pathways not previously investigated. Significantly upregulated pathways in IL-33^+^ stromal cells involved angiogenesis, cell-to-cell signaling, and morphogenesis of epithelium, supporting previous observations. Moreover, our analysis revealed involvement in axon development and axonogenesis, suggesting a potential role in neuronal development. Genes associated with these pathways, such as NGF, semaphorins, and SLIT genes, underscore the stromal cells’ contribution to neurogenic processes. The communication between stromal cells and neurons appears to be reciprocal as evidenced by the expression of neuropeptide receptors, such as *Vipr2* on stromal cells. Nevertheless, further investigations are needed to unravel the intricate roles of stromal-neuro-interactions within the intestine, offering new ways for understanding intestinal physiology and pathology.

### VIP promotes IL-33 expression in intestinal stromal cells

**FIGURE 4:**
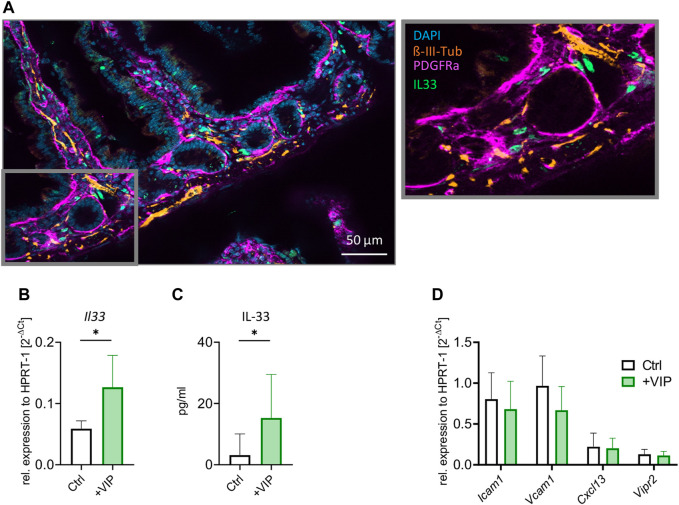
Stromal cells upregulate IL-33 in response to VIP stimulation. **(A)** Immunohistochemistry of the transverse section of the small intestine. Neurons stained in orange ( β -III-Tubulin), stromal cells in magenta (PDGFR-α) and IL-33 antibody in green. Nuclei are stained by DAPI (blue). **(B)** qPCR of *Il33* gene after stimulation of stromal cell culture with VIP, compared to Ctrl (unstimulated). **(C)** ELISA of IL-33 after stimulation of stromal cell culture with VIP, compared to Ctrl (unstimulated). **(D)** qPCR of *Icam1*, *Vcam1*, *Cxcl13,* and *Vipr2* genes after stimulation of stromal cell culture with VIP, compared to Ctrl (unstimulated). **(B–D)**: Data are representative of two experiments with three to five mice per group. Mean ± SD, Student’s *t*-test. *p < 0.05.

Our previous findings suggest a potential direct interaction between neurons and stromal cells. We aimed to investigate whether these cells co-localize in the intestine and to assess the possible involvement of neuropeptides in IL-33 regulation. As depicted in Figure 4A, our results reveal a close co-localization of IL-33^+^ cells with neurons, particularly in the lamina propria. The neuropeptide VIP has been associated with type 2 immune responses, described to potentiate the activation of immune cells and release of effector cytokines (Nussbaum et al., 2013; Talbot et al., 2015; Pascal et al., 2022). Furthermore, we observed an upregulation of *Vipr2* receptor within the IL-33^+^ stromal cell subset, with notable high expression levels compared to other neuropeptide receptors (Supplementary Figure S7B). To investigate the potential involvement of VIP in regulating the alarmin IL-33 in stromal cells, we performed primary murine intestinal stromal cell cultures (Supplementary Figure S7C,D), exposed the nearly confluent cultures to VIP overnight and determined IL-33 expression on mRNA and protein level. Indeed, we could observe a significant increase in *Il33* gene expression following VIP stimulation (Figure 4B). The stimulated stromal cells also produced higher amounts of IL-33 protein, as detected by ELISA (Figure 4C). Additionally, we investigated whether VIP stimulation of stromal cells could influence the expression of cell adhesion molecules, including ICAM-1 and VCAM-1. Moreover, we assessed *Cxcl13* and *Vipr2* expression (Figure 4D), as CXCL13 was shown to be expressed by stromal cells and play a pivotal role in recruiting B and T cells to lymphoid follicles (van de Pavert et al., 2009). Our findings suggest that VIP does not play a role in regulating cell-adhesion molecules, the chemokine *Cxcl13* or *Vipr2* expression. These findings unveil novel insights into the regulation of IL-33 by VIP and neuronal interactions with stromal cells, contributing to a deeper understanding of IL-33 biology and its potential implications for immune regulation in the intestine.

## Discussion

The intestine represents a dynamic environment where diverse cellular interactions dictate tissue homeostasis and immune surveillance. Among the broad array of cells populating the intestinal milieu, stromal cells have emerged as pivotal regulators, far beyond structural support. While prior research has highlighted the importance of stromal cells in ECM remodeling, tissue repair, and immune modulation, the precise mechanisms underlying their diverse functions remain incompletely understood. Of particular interest is the alarmin IL-33, recognized for its pleiotropic effects on immune cells and tissue homeostasis. However, the cellular source of IL-33 within the intestine has remained elusive. Herein, we present a comprehensive analysis elucidating the pivotal role of intestinal stromal cells in IL-33 secretion and unveil novel insights into their involvement in stromal-neuronal crosstalk. Using different techniques, we identified robust expression of *Il33*, implicating stromal cells as the predominant source of this cytokine within the gut microenvironment. We employed different methods in order to unequivocally examine the source of the IL-33 cytokine in the small intestine. Our investigation, utilizing IL-33-eGFP mice, revealed that both IL-33-GFP and *Il33* mRNA expression were predominantly observed in stromal cells rather than epithelial or immune cells (Figures 1A–D). The majority of intestinal stromal cells in our isolation were found to be IL-33-GFP positive. Although IL-33 was shown to be expressed by macrophages and DCs under certain conditions (Furukawa et al., 2017; Kinchen et al., 2018; Muhl et al., 2020), we failed to detect IL-33 expression over background in the small intestinal immune cells investigated from naïve mice, which could be due to steady state conditions or brightness of the reporter gene expression (Figure 1C). Nevertheless, we could demonstrate that IL-33-GFP^+^ stromal cells are able to promote ILC2 blasting in a culture experiment (Figures 1F,G). However, additional experiments are required to investigate which molecules mediate the ILC2—stromal cell interaction.

Moreover, our FACS phenotyping and transcriptomic profiling fortifies the diverse repertoire of stromal cell functions, ranging from tissue growth and remodeling to immune modulation. Current understanding suggests that tissue homeostasis relies on interactions between epithelial and stromal cells, particularly pericryptal stromal cells. These cells are believed to modulate the activity of the BMP pathway by producing ligands and inhibitors (He et al., 2004; Medema and Vermeulen, 2011). Using FACS analysis, we observed significant differences in the expression of CD81 between IL-33^+^ and IL-33^-^ stromal cells. *Cd81* was also found to be higher expressed on mRNA level (log2FC 4, data not shown). This suggests a functional specialization within the stromal cell population. Additionally, our data revealed a significant enrichment of genes linked to WNT and BMP signaling, implying a pivotal role for IL-33^+^ stromal cells in maintaining epithelial homeostasis. Comparing our results to single-cell sequencing of colonic fibroblasts from Muhl et al., we identified similarities with Tnc^−^ CD34^+^ cells located deeper, in the lamina propria and expressing WNT ligands (Muhl et al., 2020). Our IL-33^+^ stromal cells also showed enrichment for *Cd34* and other genes co-expressed in this cluster, such as *Fzd1* and *Fzd4* and *Sfrp1*. Additionally, the *Foxl1* transcription factor, which is enriched in the apical fibroblasts associated with intestinal development, was downregulated in our IL-33^+^ subset, indicating a commonality with lamina propria colonic fibroblasts (Benias et al., 2018; Muhl et al., 2020). One antagonist of the BMP signaling, *Grem1*, (Gremlin1) was demonstrated to play a pivotal role in organogenesis and differentiation, signaling through mechanisms independent of BMP, such as vascular endothelial growth factor receptor 2 (VEGFR2), SLIT proteins, and fibrillin (Dutton et al., 2019). Overexpression of *Grem1* has been associated with various cancers, including colorectal cancer (CRC) (Li et al., 2017). Pærregaard et al. recently described a population of stromal cells called trophocytes, located near the crypts in intestinal tissues and implicated in maintaining sub-crypt stem cells. This subpopulation of fibroblasts was shown to co-express CD81, CD34 and Grem-1. This suggests that a subpopulation (CD81^+^) of IL-33^+^ stromal cells might play a crucial role in supporting the crypt architecture and be similar to the defined trophocyte population (Paerregaard et al., 2023).

Furthermore, our data highlight the immunoregulatory capacity of stromal cells, as evidenced by the expression of pro-inflammatory cytokines, such as IL-1β, IL-6, or the cytokine receptors IL-17ra or IL-31R, along with chemokines including CCL2, CCL5 and TLR4. This underscores their pivotal role in shaping the local immune microenvironment. IL-17RA was shown to be highly expressed in stromal cells, including mesenchymal stem cells in humans and mice (Mojsilovic et al., 2011; Benias et al., 2018). In adipose tissues, Kohlgruber et al. revealed Pdpn^+^ and PDGFR-α^+^ stromal cells to be responsive to IL-17A. Together with TNF, these cytokines increased cell numbers and *Il33* expression, respectively, *in situ* (Kohlgruber et al., 2018). These data, together with our study, would suggest a model in which IL-17A through the upregulated *Il17ra* could possibly regulate *Il33* in intestinal stromal cells. Notably, IL-1 has been implicated in gastrointestinal inflammation, and shown to promote colitis and tumor development. While IL-1 is predominantly associated with immune cells, recent studies have revealed its interactions with epithelial cells. For instance, Wang et al. found that IL-1 can interact with epithelial cells, promoting mucin secretion by goblet cells and contribute to the excessive mucin production in the colon (Wang et al., 2023b). Moreover, neurons and glial cells express receptors for the inflammatory cytokines IL-1ß, IL-6, and IL-31. In particular, IL-1ß and IL-6 were shown to act at the neuronal level within both the peripheral and central nervous systems. For instance, IL-1β and IL-6 have been observed to depolarize guinea pig enteric neurons, leading to action potential firing, an effect mitigated by IL-1 receptor antagonists (Xia et al., 1999). Additionally, recent findings suggest that glial cells in mice contribute to intestinal inflammation by activating macrophages and polarizing them toward a pro-inflammatory state, which was amplified by proinflammatory IL-1β during a mouse colitis model (Grubisic et al., 2020). Furthermore, the cytokine IL-31 was described to connect the immune system and the peripheral nervous system. Studies have confirmed the expression of its receptor IL-31RA and signaling in a subset of murine and human DRG neurons, shedding light on its effects on sensory neurons, also including its role in itch induction (Sonkoly et al., 2006; Cevikbas et al., 2014).

In addition to their canonical functions, our study provides insights into less explored functionalities of intestinal stromal cells, implicating their involvement in stromal-neuronal crosstalk. We identified the expression of various neuropeptide receptors on intestinal stromal cells, including *Npy1r*, *Chrm3*, and *Vipr2*, suggesting their potential role as mediators of communication between the stromal cells and the nervous system. Moreover, the expression of semaphorins, SLIT genes and NTRK genes by stromal cells suggests their involvement in axonogenesis and nervous system development. These findings underscore the intricate interplay between stromal cells and neuronal elements within the intestinal microenvironment, highlighting their collective contribution to gut homeostasis and neuro-immune regulation. While the classical role of stromal cells has been associated with providing structural support and maintaining tissue integrity, emerging evidence suggests their involvement in neurogenesis (Shariati et al., 2020; Laloze et al., 2023).

Finally, our study has uncovered a previously unknown pathway whereby IL-33 expression can be regulated through the VIP-VIPR2 axis, as shown on the mRNA and protein level (Figures 4B,C). This novel finding adds to our understanding of the intricate mechanisms underlying IL-33 regulation and highlights the potential significance of the VIP-VIPR2 axis in modulating immune responses in the intestine. Interestingly, IL-33 and VIP are both stimulators of ILC2s expressing ST2 and VIPR2 (Puttur et al., 2019; Pascal et al., 2022; Topczewska et al., 2023). Our data suggest that VIP, in addition to the direct effects on ILC2, could booster IL-33 production in stromal cells, adding an additional layer of complexity to the crosstalk between stromal cells, neurons, and immune cells.

Nevertheless, further experiments are needed to fully understand and verify stromal-neuro crosstalk and its overall role in the intestinal homeostasis and neuronal development, as our work concentrated primarily on transcriptional profiling.

In conclusion, our study elucidates the central role of intestinal stromal cells in maintaining gut homeostasis and reveals their involvement in stromal-neuronal crosstalk. By identifying stromal cells as the primary source of IL-33 within the intestine and delineating their diverse functional repertoire, we provide novel insights into the complex cellular interactions governing intestinal physiology, further highlighting the potential significance of the VIP-VIPR2 axis in the regulation of IL-33. This comprehensive characterization of intestinal stromal cells not only advances our understanding of gut biology but also holds implications for the development of targeted therapeutic strategies aimed at modulating stromal cell function in various intestinal disorders.

### Experimental procedures

#### Mouse strains

C57BL/6 mice were purchased from Janvier. B6 (129S4)-*Il33* ^tm1.1Bryc^/J (IL-33-eGFP) mice were purchased from the Jackson Laboratory and bred locally at Charité. Sex and age-matched animals were used for experiments if not otherwise indicated. Males and female mice were used ranging between 8–12 weeks. We did not use randomization to assign animals to experimental groups. All animal experiments were approved and are in accordance with the local animal care committees (Lageso Berlin).

### Cell isolation

Small intestine was removed, cleaned from remaining fat tissue and washed in ice-cold PBS. The small intestine was opened longitudinally and washed in ice-cold PBS. For stromal cell isolation: dissociation of epithelial cells was performed by incubation on a shaker at 37°C in DMEM (Gibco) containing 0.1% BSA (Sigma-Aldrich) and EDTA (5 mM; Roboklon) two times for 20 min. After each step, samples were vortexed and the epithelial fraction discarded. Afterwards, remaining tissue was cut into small pieces and enzymatic digestion was performed two times for 30 min using collagenase II (1 mg/mL; Sigma-Aldrich), 10% FCS, HEPES (15 mM; Gibco) and DNase I (100 μg/mL; Sigma-Aldrich) in DMEM on a shaker at 37°C. Stromal cells were further enriched by 30/70 Percoll gradient centrifugation (GE Healthcare) (Kinchen et al. Cell 2018).

For epithelial cell isolation: Epithelial cells were dissociated by incubation on ice in DPBS (Gibco) containing EDTA (30 mM; Roboklon) and DTT (1.5 M; AppliChem) for 20 min and in a second step, by incubation in DPBS and EDTA (30 mM; Roboklon) for 10 min at 37°C on a shaker. The supernatant containing the epithelial cells was centrifuged and the cells were resuspended in HBSS (Sigma-Aldrich) and dispase (0.5 U/mL) and after centrifugation resuspended in FCS containing 100 μg/mL DNase I. The mixture was passed through a 70 μm strainer and centrifuged.

For immune cell isolation: Small intestine was removed, cleaned from remaining fat tissue and washed in ice-cold PBS. Peyer’s patches were discarded, small intestine was opened longitudinally and washed in ice-cold PBS. Dissociation of epithelial cells was performed by incubation on a shaker at 37°C in HBSS (Sigma-Aldrich) containing 10 mM Hepes (Gibco) and 5 mM EDTA (Roboklon) two times for 15 min. After each step, samples were vortexed and the epithelial fraction discarded. Afterwards, remaining tissue was chopped into small pieces and enzymatic digestion was performed using dispase (0.5 U/mL; Corning), collagenase D (0.5 mg/mL; Roche) and DNaseI (100 μg/mL; Sigma-Aldrich). Leukocytes were further enriched by Percoll gradient centrifugation (GE Healthcare). Spleens was chopped and incubated in RPMI 1640 medium (Gibco) supplemented with 1% BSA (Sigma-Aldrich), collagenase II (1 mg/mL; Sigma-Aldrich) and DNaseI (100 μg/mL) for 20 min on a shaker at 37°C. Afterwards, cells were dissociated using a Pasteur pipette, and filtered through a 70 μm cell strainer. Red cell lysis was performed in ACK lysis buffer for 3 min.

### Flow cytometry and cell sorting

Dead cells were routinely excluded with SYTOX Blue Dead Cell Stain (Thermo Fisher Scientific). Single cell suspensions were incubated on ice with anti-CD16/CD32 antibody and the following conjugated antibodies in PBS (Ca2^+^ and Mg2^+^-free, Sigma-Aldrich). If indicated, lineage-positive cells were excluded by staining for CD3e (145-2C11 or 500A2), CD5 (53–7.3), CD19 (1D3 or 6D5), FcεRIα (Mar-1) and Ly6G (1A8). For surface staining the following antibodies were used: CD11b (M1/70), CD11c (N418), CD127 (A7R34), CD4 (GK1.5 and RM4-5), CD45.2 (104), CD64 (X54-5/7.1), CD8a (53–6.7), CD81 (Eat-2), CD90.2 (53–2.1), F4/80 (BM8), KLRG1 (2F1 or MAFA), Ly6G (1A8), NK1.1 (PK136), Sca-1 (D7), SiglecF (E50-2440), ST2 (RMST2-33), FcεRIα (MAR-1), CD326 (Ep-Cam) (G8.8), CD31 (390), CD140a (PDGFR- α ), I-A/I-E (MHC class II) (M5/114.15.2). All antibodies used in flow cytometry were purchased from eBioscience, Biolegend or BD Biosciences if not otherwise indicated. All flow cytometry experiments were acquired using a custom configuration Fortessa flow cytometer and the FACS Diva software (BD Biosciences) and were analyzed with FlowJo V9.9.3 or V10.6.2 software (TreeStar) or sort-purified by using a custom configuration FACSAria cell sorter (BD Biosciences).

### Quantitative real-time PCR

Sort-purified cells were homogenized in Trizol (Thermo Fisher Scientific) and stored at −80°C. RNA was extracted with chloroform and RNA concentration was determined using a Nanodrop 2000 spectrophotometer (Thermo Fisher Scientific). Reverse transcription of total RNA was performed using the High Capacity cDNA Reverse Transcription kit according to the protocol provided by the manufacturer (Thermo Fisher Scientific). Reaction was detected on a QuantStudio 5 Real-Time PCR (Thermo Fisher Scientific) using Taqman Gene Expression Assay (Applied Biosystems) with *Epcam* (Mm00493211_m1), *Il33* (Mm00505403_m1), *Pdgfra* (Mm00440701_m1), *Vipr2* (Mm01238618_g1) or SYBR Green Master Mix using *Il33* (forward: 5′-TGA​GAC​TCC​GTT​CTG​GCC​TC -3′, reverse: 5′-CTC​TTC​ATG​CTT​GGT​ACC​CGA​T-3′), *Vcam1* (forward: 5′-TTG​GGA​GCC​TCA​ACG​GTA​CT-3′, reverse: 5′-GCA​ATC​GTT​TTG​TAT​TCA​GGG​GA-3′), *Icam1* (forward: 5′-TCC​GCT​ACC​ATC​ACC​GTG​TAT-3′, reverse: 5′-TAG​CCA​GCA​CCG​TGA​ATG​TG-3′), *Cxcl13* (forward: 5′-ATA​TGT​GTG​AAT​CCT​CGT​GCC​A-3′, reverse: 5′- GGG​AGT​TGA​AGA​CAG​ACT​TTT​GC -3′). Gene expression was normalized to the housekeeping gene *Hprt1* (Mm00446968_m1) for Taqman and *Hprt1* (forward: 5′-GAT​ACA​GGC​CAG​ACT​TTG​TTG​G-3′, reverse: 5′-CAA​CAG​GAC​TCC​TCG​TAT​TTG​C-3′) for SYBR Green.

### Cell culture

Stromal cells were sort-purified (as live CD45^−^, Epcam^−^, CD31^−^ and PDGFR- α ^+^ and Sca-1^+^) and 12.000 cells/well were seeded in a 96-well plate in DMEM containing 10% FCS, 1% NEAA and 1% Pen/Strep. Stromal cells were incubated for 7 days to reach confluency at 37°C and 5% CO_2_. On day 7, 8.000–10.000 sort-purified ILC2s (as live CD45^+^, Lin^−^, NK1.1^-^, CD127^+^ and KLRG1^+^ for small intestine) were added per well in DMEM with high glucose supplemented with 10% FCS, 10 mM Hepes, 1 mM sodium pyruvate, non-essential amino acids, 80 μM 2-Mercaptoethanol, 2 mM Glutamine, 100 U/mL Penicillin and 100 μg/mL Streptomycin (all from Gibco). ILC2s only were seeded as controls. The culture was supplemented with a cocktail of IL-7 only or IL-7 and IL-33 (Biolegend or R&D systems, 20 ng/mL and 10 ng/mL, respectively). After 4 days of co-culturing, supernatant was collected and ILC2s were stained with Sytox Blue Dead Cell Stain. The data was acquired by a BD LSRFortessa X-20 Flow Cytometer and analyzed by FlowJo v10.6.0.

### Histology and immunofluorescence microscopy

For immunofluorescence staining, the small intestine of WT mouse was cut open longitudinally, the feces were washed off and swiss-rolls were formed. The tissue was fixed in 4% PFA at 4°C and soaked in 30% sucrose until tissue embedding with TissueTEK. After cryosectioning, the slides were stored at −80°C until staining. Cryosections were dried and then fixed using acetone (Carl Roth) at −20°C. Sections were permeabilized with 0.5% Saponin (Sigma-Aldrich) in PBS and blocked with 10% serum and stained with rabbit anti-mouse β -III-Tubulin (T2200) for neurons, followed by donkey anti-rabbit antibody coupled to Alexa Fluor 555 (Thermo Fisher Scientific) and rat anti-mouse PDGFR-α (APA5) followed by donkey anti-rat Alexa Fluor 647 (Thermo Fisher Scientific). IL-33 was captured via goat anti-mouse IL-33 (R&D systems, AF3626-SP) followed by donkey anti-goat Alexa Fluor 488 (Thermo Fisher Scientific). Nuclei were counterstained with DAPI (Thermo Fisher Scientific). Images were captured on a Zeiss Axio Observer seven microscope and analysed with Zen software (Zeiss). After culturing the stromal cells for 4 days in medium, cells were fixed as described above and imaged directly in the well.

### Bulk-RNA-sequencing

Stromal cells were sort purified as live CD45^−^ Epcam^−^ CD31^−^ PDGFR- α ^+^ Sca-1^+^ from the small intestine of IL-33-eGFP mice. Cells were sorted into Trizol and isolated using miRNeasy Plus Micro kit (Qiagen) according to manufacturer’s protocol. RNA-seq libraries were prepared by the MDC BIMSB Core Bioinformatic Facility using Takara SMART-Seq Ultra Low Input Kit (Takara Bio). Sequencing was carried out on a NovaSeq6000 (Illumina), yielding 100 bp single-end reads. Raw sequencing reads were mapped to the mouse genome (mm10) with STAR (Dobin et al., 2013) version 2.7.11a using default parameters. Reads were assigned to genes with FeatureCounts (Liao et al., 2014) with the parameters: t exon -g gene_id, gene annotation was performed on GRCm38 (mm10). The differential expression analysis was performed with DESeq2 (Love et al., 2014) version 1.22.1 using default parameters, IL-33-eGFP^+^ vs. IL-33-eGFP^-^. We kept genes having a minimum of 30 reads in all the samples. The data presented in the study are deposited in the SRA repository, accession number PRJNA1139407.

### Primary murine stromal cell culture and stimulation with VIP

Stromal cell cultures from the small intestine were established using an adapted protocol from Lin et al. (Lin et al., 2023). Briefly, the small intestine was carefully excised, longitudinally cut, washed twice in fresh PBS and incubated for 20 min in PBS containing 10 mM EDTA and 0.5 mM dithiothreitol (DTT) at 37°C. Subsequently, the tissue was transferred to ice-cold PBS and vigorously shaken to remove the epithelium and intestinal crypts. This step was repeated 4–5 times until a clear supernatant was obtained. Then the remaining tissue was cut into tiny pieces and incubated in digestion buffer composed of calcium and magnesium-free HBSS supplemented with Liberase TL (1 unit/mL, Roche), and DNase I (1 mg/mL, Sigma Aldrich) for 1 h on a shaker at 37°C and pipetting every 10 min. Every 20 min the supernatant containing the digested fraction was collected and new digestion buffer was added for the remaining time. The digested fractions were collected in stromal cell medium composed of Advanced DMEM/F12 supplemented with 10% FCS, 10 mM HEPES (Life Technologies), 2 mM L-Glutamine (Thermo Fisher Scientific), 100 U/mL Penicillin/Streptomycin (Gibco) and 10 µM Y-27632. The collected fractions were then filtered through a 70 μm cell strainer and centrifuged at 500 *g* for 5 min at 4°C. The cells were then seeded on 6-well plates in stromal cell medium (Y-27632 was only added during the first 2 days). Once cells reached 80%–90% confluency after 4–5 days they were split and in a 96-well plate. Cells were allowed to attach for 24 h and then stimulated with 1 µM VIP for 24 h. Finally, RNA was isolated using Trizol for downstream applications and supernatants and cell lysate were taken for ELISA.

### ELISA

Cell lysate from stromal cell culture was used for detection of IL-33 after two freeze (−80°C) and thaw (37°C) cycles for 20 min under each condition. IL-33 protein was measured using the Mouse/Rat IL-33 Quantikine ELISA Kit (R&D systems) according to manufacturer’s protocol and detected on a Tecan Infinite M Plex plate reader at 450 nm with reference wavelength at 570 nm within 10 min after addition of the stop solution. IL-33 protein concentrations were calculated based on the acquired standard curve.

### Statistical analysis

Data is plotted showing the mean ± standard deviation. *p* values of data sets were determined by unpaired two-tailed Student’s *t*-test with 95% confidence interval. If equal variances could not be assumed, Welch test was performed. Ordinary one-way ANOVA with Tukey’s multiple comparisons test was used to analyze several groups. Normal distribution was assumed. Before mentioned statistical tests were performed with Graph Pad Prism V9 software (GraphPad Software, Inc.). (*p < 0.05; **p < 0.01; ***p < 0.001; ****p < 0.0001 and ns, not significant).

## Data Availability

The datasets presented in this study can be found in online repositories. The names of the repository and accession number can be found in the article’s experimental procedures.
