# Supplementary figures and images for: Transcriptional profiling identifies IL-33-expressing intestinal stromal cells as a signaling hub poised to interact with enteric neurons
Source: Front Cell Dev Biol. 2024 Aug 1;12:1420313. doi: 10.3389/fcell.2024.1420313 (PMC11325031; doi:10.3389/fcell.2024.1420313)

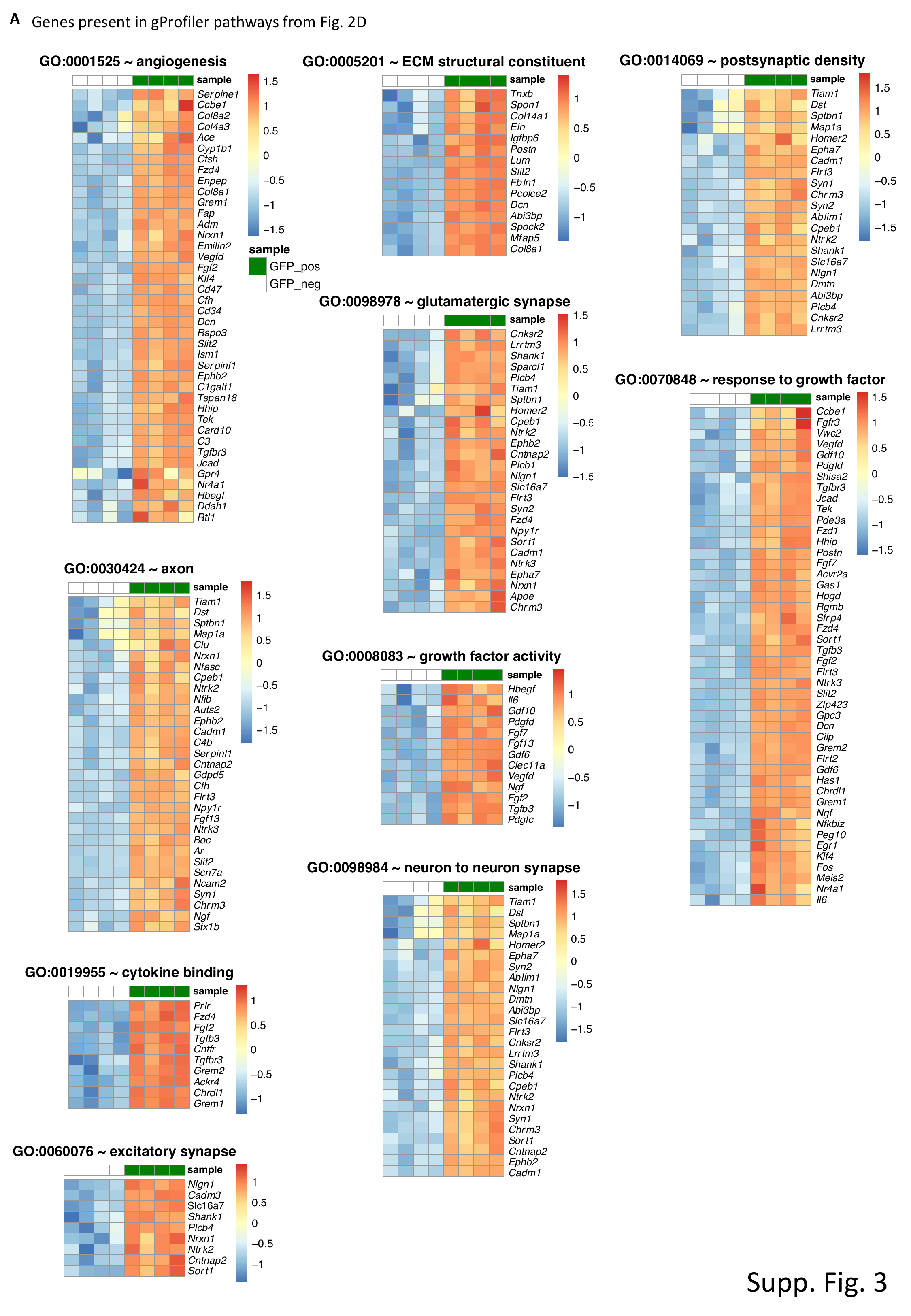

Supplement: Supplementary file 1 [file Image3.TIFF]

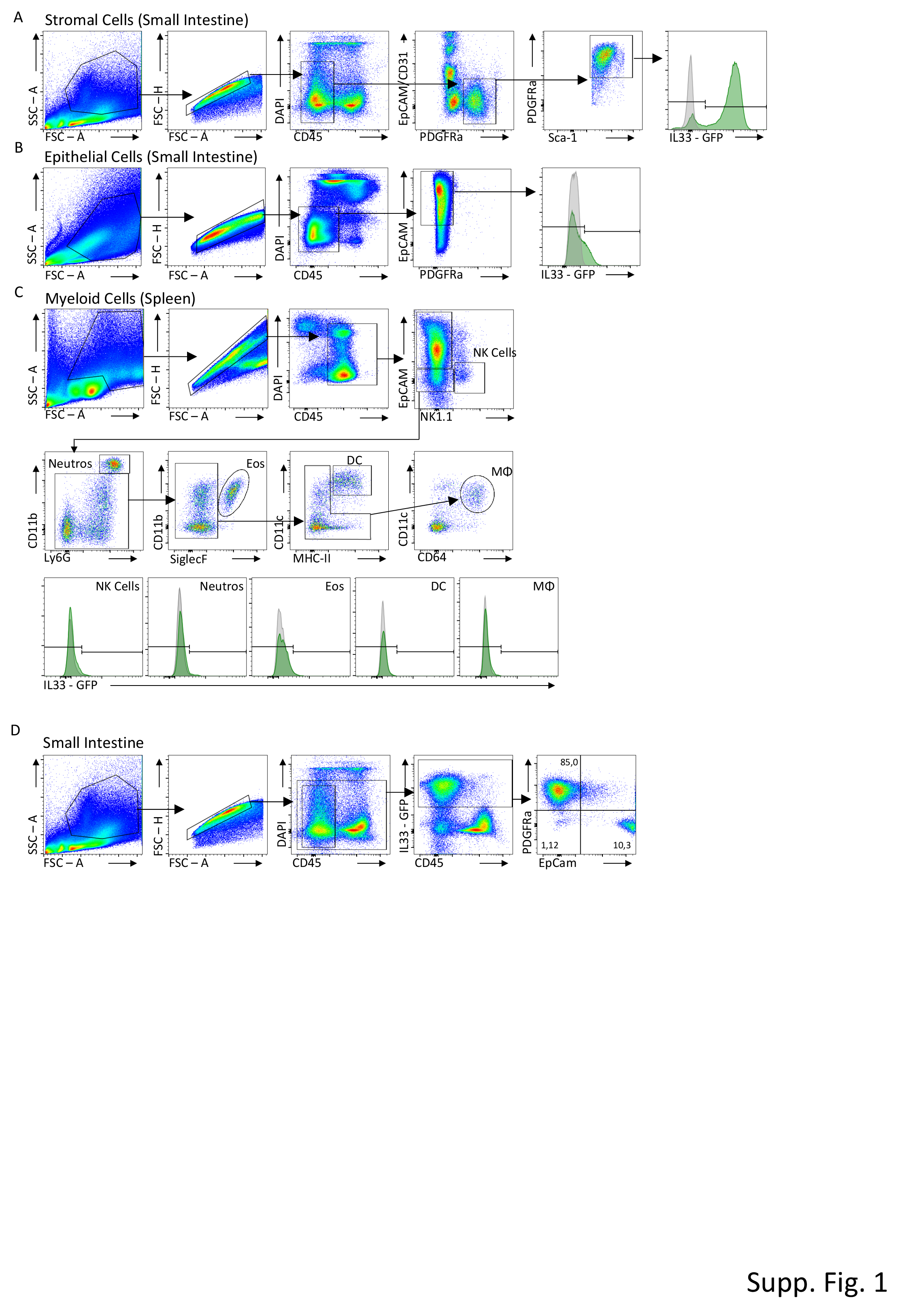

Supplement: Supplementary file 2 [file Image1.TIFF]

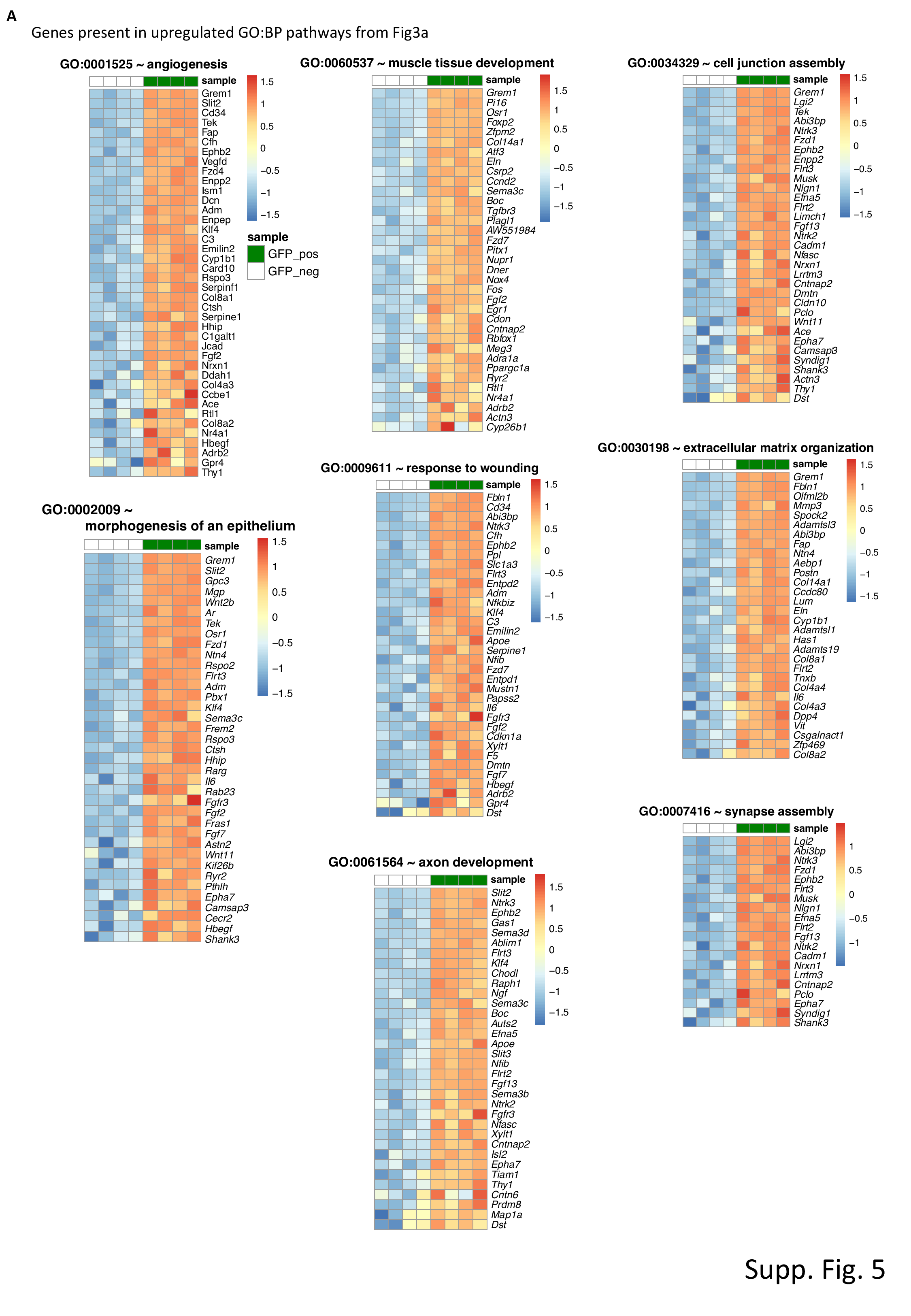

Supplement: Supplementary file 3 [file Image5.TIFF]

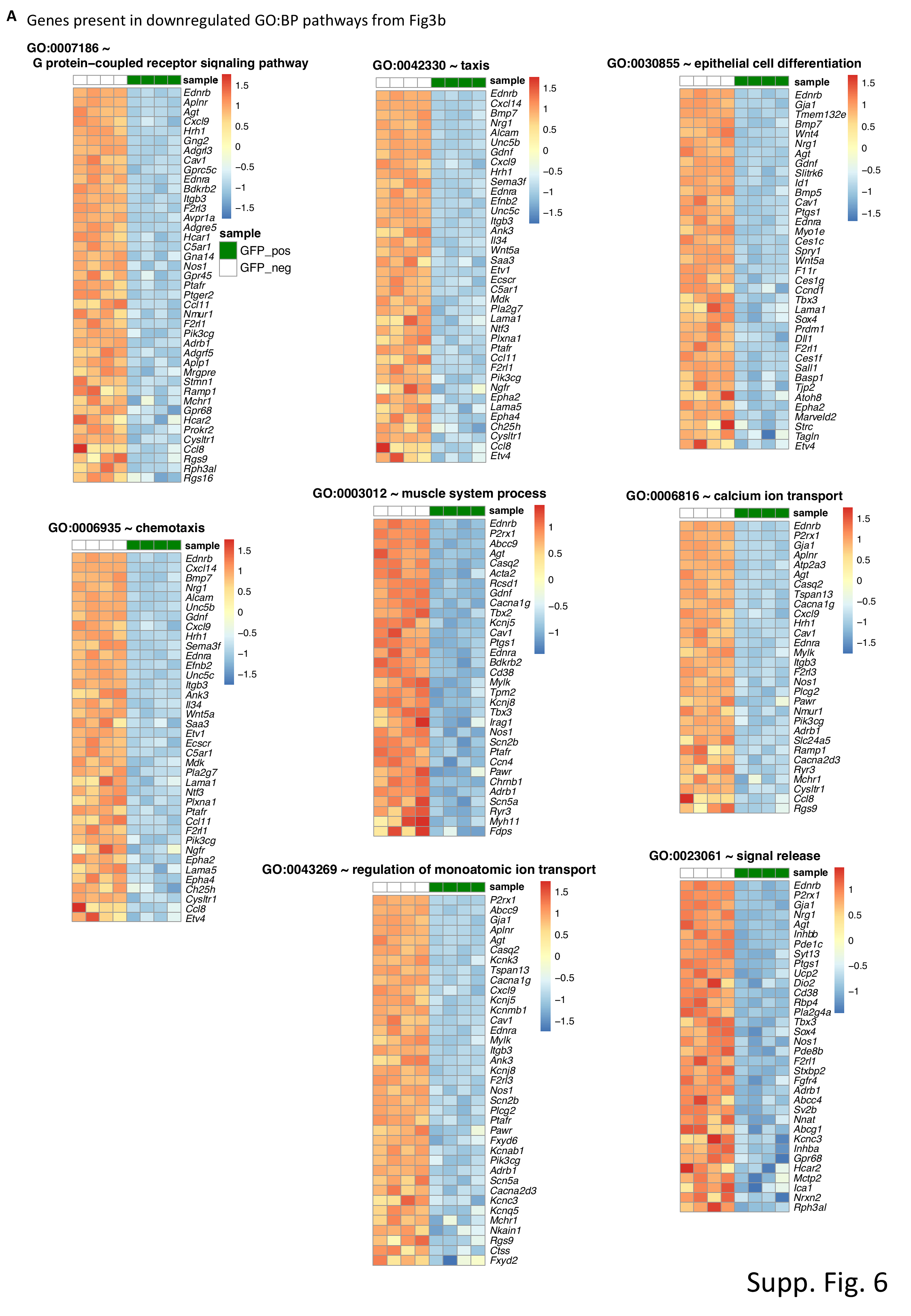

Supplement: Supplementary file 4 [file Image6.TIFF]

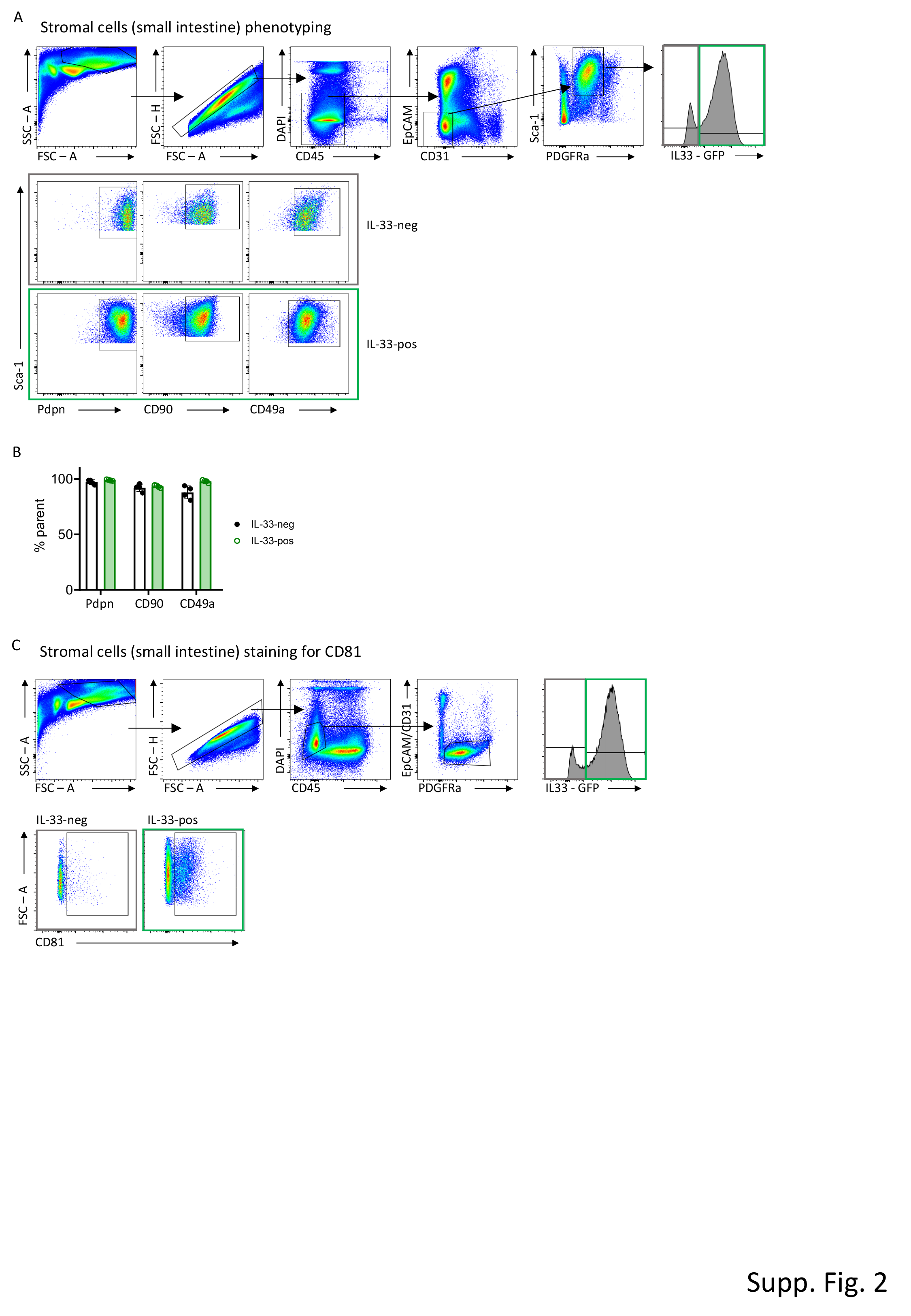

Supplement: Supplementary file 5 [file Image2.TIFF]

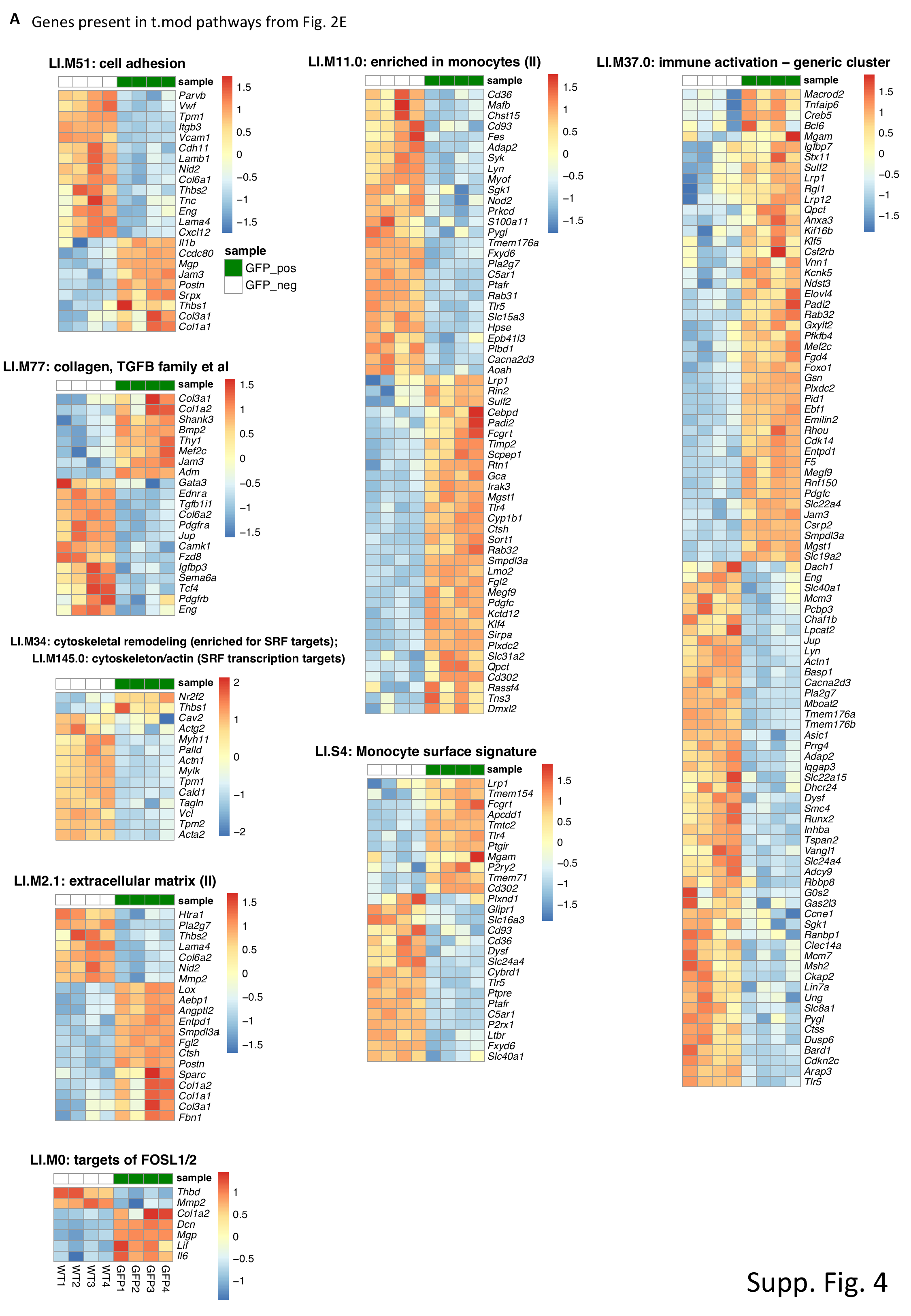

Supplement: Supplementary file 6 [file Image4.TIFF]

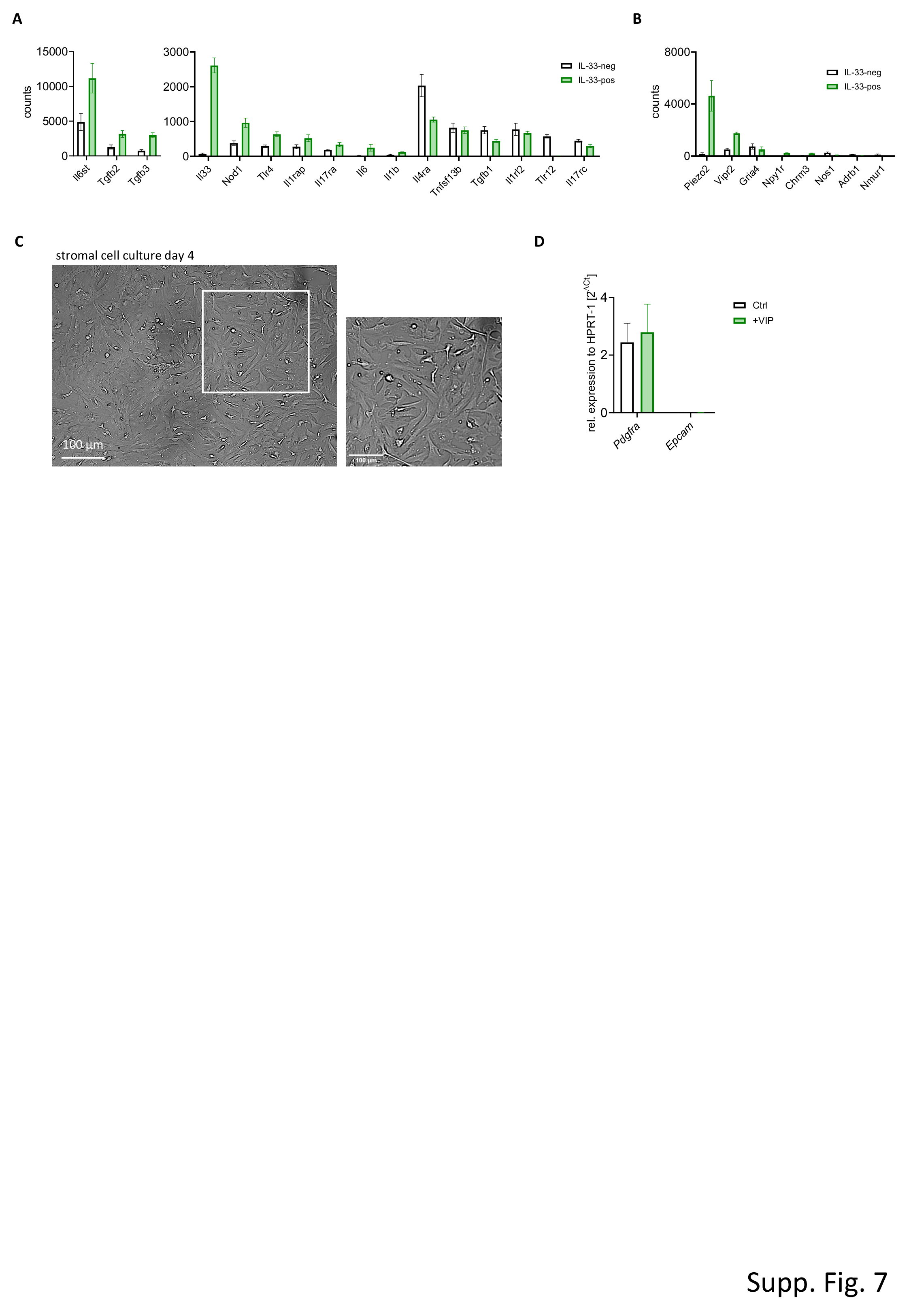

Supplement: Supplementary file 7 [file Image7.TIFF]
